# Gynecological Cancers Translational, Research Implementation, and Harmonization: Gynecologic Cancer InterGroup Consensus and Still Open Questions

**DOI:** 10.3390/cells8030200

**Published:** 2019-02-26

**Authors:** Marina Bagnoli, Ting Yan Shi, Charlie Gourley, Paul Speiser, Alexander Reuss, Hans W. Nijman, Carien L. Creutzberg, Suzy Scholl, Anastassia Negrouk, Mark F. Brady, Kosei Hasegawa, Katsutoshi Oda, Iain A. McNeish, Elise C. Kohn, Amit M. Oza, Helen MacKay, David Millan, Katherine Bennett, Clare Scott, Delia Mezzanzanica

**Affiliations:** 1 Department of Research, Fondazione IRCCS Istituto Nazionale dei Tumori di Milano, 20133 Milan, Italy; marina.bagnoli@istitutotumori.mi.it; 2 Department of Obstetrics and Gynecology, Zhongshan Hospital, Fudan University, Shanghai 200032, China; tyshi80@163.com; 3 University of Edinburgh Cancer Research UK Centre, MRC IGMM, Edinburgh EH4 2XU, UK; Charlie.Gourley@ed.ac.uk; 4 Department of Gynaecologic Oncology, Medical University Vienna, General Hospital Vienna, 1090 Wien, Austria; pspeis54@gmail.com; 5 Coordinating Center for Clinical Trials, at the Philipps-University of Marburg, 35043 Marburg, Germany; alexander.reuss@kks.uni-marburg.de; 6 Department of Obstetrics & Gynecology, University Medical Center Groningen, 9700 RB Groningen, The Netherlands; h.w.nijman@umcg.nl; 7 Department of Radiation Oncology, Leiden University Medical Center, 2333 ZA Leiden, The Netherlands; c.l.creutzberg@lumc.nl; 8 Department of Drug Development and Innovation, Institut Curie, 75005 Paris, France; suzy.scholl@curie.fr; 9 European Organisation for Research and Treatment of Cancer (EORTC), 1200 Brussels, Belgium; anastassia.negrouk@eortc.org; 10 Department of Biostatistics & Bioinformatics, Roswell Park Cancer Institute, Buffalo, NY 14203, USA; brady@gogstats.org; 11 Department of Gynecologic Oncology, Saitama Medical University International Medical Center, Saitama 1397-1, Japan; koseih@gmail.com; 12 Department of Obstetrics and Gynecology, Graduate School of Medicine, The University of Tokyo, Tokyo 113-8654, Japan; katsutoshi-tky@umin.ac.jp; 13 Division of Cancer, Department of Surgery and Cancer, Imperial College London, London SW7 2AZ, UK; i.mcneish@imperial.ac.uk; 14 Clinical Investigations Branch, Cancer Therapy Evaluation Program, National Cancer Institute, Rockville, MD 20852, USA; kohne@mail.nih.gov; 15 Department of Medicine, Division of Medical Oncology & Hematology, Princess Margaret Cancer Centre, University Health Network, University of Toronto, Toronto, ON M5G 2M9, Canada; Amit.Oza@uhn.ca; 16 Division of Medical Oncology, University of Toronto/Sunnybrook Odette Cancer Centre, Toronto, ON M4N 3M5, Canada; helen.mackay@sunnybrook.ca; 17 Department of Pathology, Queen Elizabeth University Hospital, Glasgow G51 4TR, UK; david.millan59@btinternet.com; 18 Gynecologic Cancer InterGroup, Operations, Kingston, ON K7K-7A6, Canada; gcigopsasst@gmail.com; 19 Walter and Eliza Hall Institute of Medical Research, Parkville, Victoria 3052, Australia; scottc@wehi.edu.au

**Keywords:** translational studies design, gynecological cancers, biomarkers definition, precision medicine, samples collection

## Abstract

In the era of personalized medicine, the introduction of translational studies in clinical trials has substantially increased their costs, but provides the possibility of improving the productivity of trials with a better selection of recruited patients. With the overall goal of creating a roadmap to improve translational design for future gynecological cancer trials and of defining translational goals, a main discussion was held during a brainstorming day of the Gynecologic Cancer InterGroup (GCIG) Translational Research Committee and overall conclusions are here reported. A particular emphasis was dedicated to the new frontier of the immunoprofiling of gynecological cancers. The discussion pointed out that to maximize patients’ benefit, translational studies should be integral to clinical trial design with standardization and optimization of procedures including a harmonization program of Standard Operating Procedures. Pathology-reviewed sample collection should be mandatory and ensured by dedicated funding. Biomarker validation and development should be made public and transparent to ensure rapid progresses with positive outcomes for patients. Guidelines/templates for patients’ informed consent are needed. Importantly for the public, recognized goals are to increase the involvement of advocates and to improve the reporting of translational data in a forum accessible to patients.

## 1. Introduction

Current practice for gynecological cancers is based on the evidence generated by clinical trials mainly designed without translational end-points. In the era of personalized cancer therapy, the knowledge of patients’ tumor and germline molecular characteristics is becoming essential for a better definition of a trial’s inclusion/exclusion criteria and better understanding and interpretation of the results. Translational studies are often designed and performed after the corresponding clinical trial has been completed, with translational end-points not included in the original study design with the consequence that the collection of biological material is retrospective and not performed at study entry or at diagnosis. Tissue samples collected at the end of patients’ recruitment might significantly diminish the possibility of including all the intention-to-treat population in the translational analysis, thus, compromising the possibility of having the right samples to answer the right question. Guidelines and consensus on translational end-points and methodologies may improve the design of pivotal trials minimizing the impact of bias. This manuscript provides a summary of the topics discussed during the Gynecologic Cancer InterGroup (GCIG) Translational Research brainstorming day and provides an overview of the consensus process together with ongoing questions/problems that remain to be addressed.

## 2. Precision Medicine in Gynecological Cancer

What we expect from precision medicine is to contribute to our understanding of human cancer biology and to define appropriated patient populations, based on their molecular characteristics, to maximize the efficacy of personalized therapies. As a consequence, biospecimen-derived biomarkers have increasingly been incorporated into therapeutic cancer clinical trials. However, the decision to incorporate a biomarker into a clinical trial requires serious consideration of its advantages and disadvantages as well as a careful choice of the most appropriate biomarker [1,2]. In addition to the extremely important biological information that a biomarker may provide, some critical issues should be taken into consideration during translational study design.

1. Translational studies can add complexity and may impact on recruitment to the clinical trial.
2. Clear definition of the hypotheses driving correlative studies is essential.
3. The trial must be adequately designed and sufficiently powered to address the translational hypotheses.

Clear guidelines are, therefore, needed to ensure the most accurate selection of reliable biomarkers in terms of both detection and function in the clinic.

### 2.1. Biomarkers: Definitions and Applications

According to the Biomarkers Definitions Working Group [3], a biomarker is ‘a characteristic that is objectively measured and evaluated as an indicator of normal biological processes, pathologic processes, or pharmacologic responses to a therapeutic intervention’. Biomarkers can be specific cells, molecules, genes, gene products, enzymes or hormones and need to fulfill three major criteria, to be: practical, effective and reproducible.

Biomarkers have a number of applications, guiding disease prevention, diagnostic and prognostic assessment, drug target identification, and drug response (Table 1). Among the different biomarkers, the most frequently confused, in terms of applications and definitions, are prognostic versus predictive markers. *Prognostic markers* inform about a likely outcome independently of any particular treatment. *Predictive markers* are associated with benefit or lack of benefit from a specific therapy; they are of particular relevance in personalized therapy as the treatment effect is different for patients with positive or negative biomarker status. The interaction between the treatment effect and marker status can be *qualitative*, when patients positive for the biomarker benefit from the treatment but the others receive no benefit or possibly even harm, or *quantitative*, when treatment benefits all patients but by different amounts. Qualitative interaction can clearly help in guiding treatment choice, whereas in the case of quantitative interaction it is more difficult to establish whether or not the biomarker will be useful for selection of a new treatment because both patient subgroups derive some benefit from treatment.

Biomarkers can also be divided into integral, integrated, and exploratory, each having their specific requirements (Table 2). Integral biomarkers must be identified a priori for the trial to proceed (e.g., a particular biomarker status may be a pre-requisite for patient entry or required for stratification). Such biomarkers are inherent to the trial design and must be performed in real time, to underpin the conduct of the trial. Integrated biomarkers are those clearly specified to address pre-stated hypotheses. Often they are intended to identify or validate assays or markers that are planned for use in future trials. Their design should be as precise as for integral biomarkers, but the assay result is not used for eligibility or treatment assignment in the current trial. Exploratory biomarkers are only descriptive in the perspective of alternative treatments.

**Figure 1:**
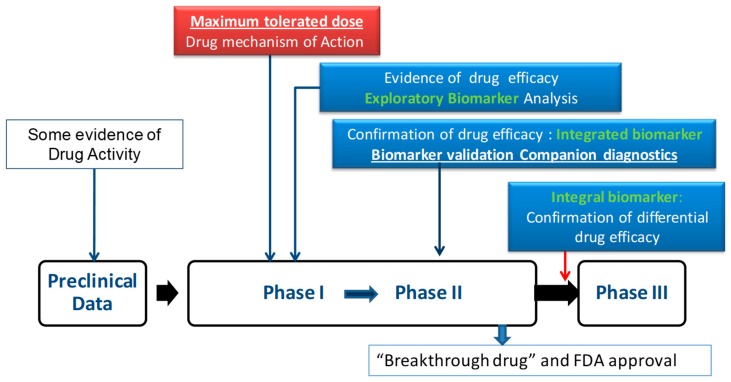
Paradigm for use and definition of biomarkers in clinical trials. Validation of a biomarker to be considered integral to a clinical trial from its pre-clinical definition. Slide courtesy of Dr. A. Oza from his presentation to the Gynecologic Cancer InterGroup (GCIG) Translational Research brainstorming day.

A biomarker, to be considered integral, needs to be validated from preclinical observation to clinical application (Figure 1). Proceeding through phase II-III trials, the first priority is to establish the efficacy of the novel agent. The identified biomarker must then have sufficient predictive power to allow patient selection. The ideal validation of a predictive biomarker is the demonstration that an agent is more effective than standard therapy in biomarker-positive patients and no more effective than standard therapy in biomarker-negative patients. This 2 × 2 approach (randomization) relies upon a positive test for interaction and prevents prognostic biomarkers from being mistaken for predictive biomarkers. In the corresponding trial design, only biomarker-positive patients will be randomized to treatment. In this situation, it is important to be confident regarding correct biomarker selection and to be aware of the potential for responsive patients to exist in the biomarker-negative group due to treatment effects not directly related to biomarker expression and/or activity.

An excellent example of biomarkers application is the ENGOT OV-16 NOVA trial [4] of maintenance therapy for High-grade Serous Ovarian Cancer (HGSOC); it included integral, integrated, and exploratory biomarkers.

**Figure 2:**
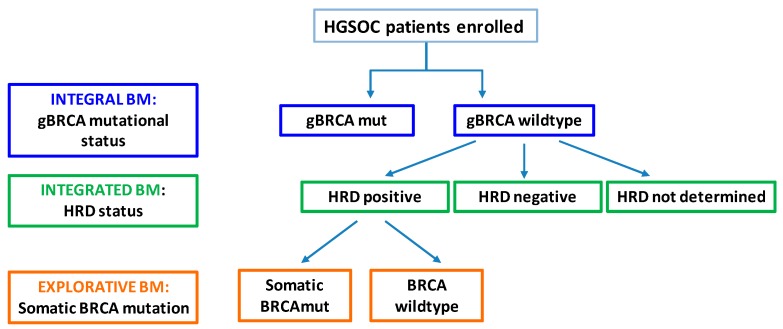
Application of integral, integrated and explorative biomarker analysis in the ENGOT-OV-NOVA16 trial design. (gBRCA: germline BRCA; mut: mutated; HRD: homologous recombination deficiency).

The integral biomarker was the detection of germline *BRCA1/2* mutation before randomization for treatment with a PARP inhibitor vs. placebo. In the *BRCA1/2* germline wild type arm, an integrated study was nested, which characterized tumors according to homologous recombination deficiency (HRD) status and allowed assessment of treatment efficacy in the HRD-positive population in terms of progression-free survival (PFS). Finally, an exploratory analysis of PFS was performed in specific subgroups of the *BRCA1/2* germline wild type cohort, defined by taking into account the HRD status (positive vs. negative) and the presence of somatic *BRCA1/2* mutation vs. *BRCA1/2* wild type in the HRD-positive cohort (Figure 2).

### 2.2. Biospecimen, Quality Control, and Validation

Identification of appropriate targets for tumor detection, therapy and prevention rely on our ability to generate high-quality patient-derived specimens whose collection, storage, handling, and processing must be well controlled to prevent assigning clinical significance to artefactual findings.

There should be a commitment to optimize quality in biomarker detection to maximize the chance of successful validation. This involves ensuring that the procedures for the entire biomarker pipeline are accurate, standardized, and reproducible (including sample collection, processing, assay, scoring system, and threshold selection). Analytical validation of an assay involves assessment of accuracy, precision, specificity, and sensitivity to ensure sufficient intra- and inter-laboratory reproducibility. Furthermore, moving from preclinical models to human patients, the assay should fit for use on clinical specimens. An approved reference standard that can be used for the development/validation of the assay is recommended.

The choice of the appropriate clinical trial strategy depends on the strength of the existing evidence for the biomarker (the biomarker credentials) and the questions being addressed (clinical endpoints). Basic designs of randomized phase III biomarker-driven trials with time-to-event end point (overall survival, disease-free survival, relapse free survival) include biomarker-enrichment and biomarker-stratified designs, with adaptive designs being increasingly incorporated (Table 3).

Advances in the *omics* era posed a considerable challenge for the definition of clinical trial strategies. Indeed, the evaluation of a targeted treatment in the early development phase required accurate selection of the patient population and led to smaller trials, resulting in smaller datasets on which to build the body of evidence necessary to support the use of a drug/therapeutic agent for a particular indication. To address this, new clinical trial designs were developed:

*Basket studies:* Patients are selected according to their molecular characteristics and biomarkers, regardless of the site of origin of their tumor. Usually, they focus on a single drug targeting a single biomarker in different tumors. An example is BRAF V600 Vemurafenib enrolling patients with different non-melanoma cancers harboring BRAF V600 mutation [5].

*Umbrella studies:* They enroll patients with a single tumor type, defined by primary anatomic site, and direct them towards different treatments according to the molecular characterization of each case. An example is the FOCUS4 study in colorectal cancer, which stratifies patients according to biomarker positivity or negativity into seven different randomizations, the last of which is for patients not yet stratified by any of the preceding biomarkers [6].

*Platform trials:* As in the case of umbrella trial, the focus is on the disease rather than on a particular type of therapy but, rather than assuming that we know which drug is appropriate for which biomarker stratum, randomization among drugs is used in the platform trial. They can be considered as an extension of adaptive trials as accumulating outcome data can be used to adjust randomization to assign the better performing treatment regimen. An example is the BioRAIDs trial on cervical cancer whose challenge was to identify potentially curative therapeutic interventions based on a rational molecular assessment leading to the design and selection of innovative drugs [7].

### 2.3. Improvement of Biomarker Development

Longitudinal collection of tissue and liquid biopsy, imaging, and robust clinical data annotation are key elements for the achievement of an integrated, personalized medicine strategy. The BriTROC-1 prospective observational study [8] involving 12 cancer centers across the UK addressed this challenge by profiling multiple samples (blood and tissue) from 300 patients with high grade serous ovarian cancer (HGSOC) during the course of their disease (at diagnosis, at the end of therapy, and upon relapse) to:

- Assess longitudinally, cancer cell clonal evolution and dynamics in cohorts or individual cancer patients and to capture heterogeneity in space and time [9].
- Guide the use of molecular-targeted therapies and provide critical insight into mechanisms of treatment failure and resistance in cancer patients.

It has to be taken into consideration that longitudinal clinical studies are complex and require dedicated personnel, expert in activities over the ‘routine’ clinical trial (e.g., coordinating multiple parallel clinic/diagnostic/biopsy appointments, entering eCase Report Forms). Liquid biopsies appear to be an attractive approach for longitudinal analyses. However, techniques, such as cell free DNA (cfDNA) analysis, that can be performed on liquid biopsy still require refinement and validation. In high-grade serous ovarian cancer, *TP53* mutation is an obvious candidate for such validation, as it is almost ubiquitously mutated and can be detected immunohistochemically and by sequencing in tumors as well as in cfDNA [10].

A further valuable example derives from the TransPORTEC consortium: An international collaborative group with multidisciplinary experts (clinical, radiation, medical and gynecological oncologists, pathologists, scientists, and statisticians) working on endometrial cancer with the aims to discover: (1) molecular prognostic factors, (2) predictors of chemotherapy efficacy, (3) immune response markers (4) new targets for therapy, and (5) driver mutations, and mechanisms of endometrial cancer development and spread. The PORTEC-1 and PORTEC-2 trials together recruited about 1000 stage I-II endometrial cancer patients with high–intermediate risk factors to evaluate the role of pelvic radiotherapy (PORTEC-1) vs. vaginal brachytherapy (PORTEC-2). Translational studies derived from PORTEC-1/2 trial biobank, incorporating comprehensive analysis of molecular factors, have led to improved risk assessment of endometrial cancer [11].

A centralized prospective biobank with well-defined methods and processes was put in place with patient consent covering the donation of tissues sample. Together with archival fixed histologic tumor material, matched tumor, and normal DNA was collected for each patient. Expert pathology review was performed to ensure adequate assessment of pathological factors, thus, facilitating the establishment of a molecular integrated risk assessment model. In the integrated model, besides the existing “big five” pathologic assessments (tumor type, grade, myometrial involvement, endocervical involvement, and lympho–vascular space invasion), immunohistochemistry for key molecules as well as molecular features, have been included [11]. These analyses allowed the identification of three molecular integrated risk profiles: (1) unfavorable, (2) intermediate, (3) favorable, reclassifying about 70% of endometrial cancer patients. In the PORTEC-3 trial, upfront expert pathology review was mandatory before the inclusion of a patient in the trial to ensure a true high-risk population [12]. The TransPORTEC biobank now contains over 400 tissue samples donated by women participating in the trial. To prepare for translational research with the PORTEC-3 trial samples, the consortium worked to put the logistics, including an application for funding, into place for international collaborative biomarker studies and to generate hypotheses for further research. As a result, a new trial, PORTEC-4, has been initiated to compare individual molecular integrated therapy with standard indications for the most appropriated adjuvant treatment.

## 3. Immunoprofiling of Gynecological Cancers: Towards a New Class of Biomarkers?

Cancer is characterized by the accumulation of genetic alterations resulting in the expression of neoantigens that could be presented on the surface of cancer cells in association with a major histocompatibility complex (MHC), thus, distinguishing them from their normal counterparts and stimulating an immune response [13,14]. Identification of the mechanisms by which tumor cells adapt to evade the anti-tumor immune response may help in rational drug development and treatment selection for personalized cancer immunotherapy [15]. Some tumors might not be infiltrated by immune cells (so-called ‘cold tumors’) and in the cases where immune cell infiltration is present; three major players could contribute to their activity: the type of immune infiltrate, tumor microenvironment-specific factors, and antigen presentation machinery. Understanding and defining the immune profile of a patient’s tumor is key to exploiting it.

### 3.1. Tumor-Infiltrating Immune Cells

The association of tumor-infiltrating lymphocytes (TIL) with improved ovarian cancer prognosis was initially described by G. Coukos and colleagues [16] and confirmed by a meta-analysis of ten studies including 1815 OC patients [17]. However, to be effective, immunotherapy-based strategies need to accurately characterize the different TIL populations to identify which population should be targeted or stimulated. Indeed, mainly cytotoxic TIL were those associated with prolonged survival [18], while the presence of high numbers of intratumoral immunosuppressive regulatory T cells, inhibiting the T cell-mediated anti-tumor response, was associated with worse outcome [19]. The evidence that the local (immune microenvironment) and systemic host T cell immune profile could be significantly different [20] highlighted the relevance of considering both profiles when designing immunotherapeutic strategies.

### 3.2. Tumor-Specific Factors

Mechanisms that limit immune responses toward a cancer, essentially act by inhibiting cytotoxic T cell effects and there are a number of immune-checkpoints which regulate interactions between T cells, antigen presenting cells, cells of the innate immune system, such as macrophages, and tumor cells. Among the immune-checkpoints, PD-L1 expression on tumor cells represents a major immune evasion strategy for many cancers. Indeed, binding of the check-point molecule, PD-1, expressed by T cells, to its ligand PD-L1, expressed by tumor cells, prevents T cell receptor activation [21]. Therefore, antibodies directed against PD-1 expressed on T cells and/or antibodies directed against PD-L1 expressed on tumor cells, preventing the ligand-receptor binding could ‘release the brake’ and permit T cell activation.

In ovarian cancer patients, the prognostic role of PD-L1 expression on tumor cells appears to be contradictory [22,23]. In this complex setting, identifying ovarian cancer patients with a high number of the specific T cell subpopulation that could mostly respond to stimulation by acting on checkpoint molecules, could be a strategy to identify those patients who could most benefit from this type of treatment [24]. It would also be very helpful to understand which histopathological or molecular (clear cell carcinoma or BRCA1-mutant HGSOC) subsets of ovarian and other gynecological cancers (mismatch repair (MMR) deficient endometrial cancer) are primed to respond to immune checkpoint inhibitors.

### 3.3. Tumor Antigenicity

Neo-antigens are non-self-antigens, typically derived from tumor-specific somatic mutations and can be targeted by the host immune system. Advances in sequencing technology, which allow the direct identification of tumor neoantigens, can now be exploited for immune therapy. Hyper-mutated cancers such as melanoma, lung cancer or tumors with MMR deficiency, harboring a high number of tumor-specific neo-antigens, stimulate the recruitment of TIL and have high response rates to treatment with immune checkpoint inhibitors. Ovarían cancer typically harbors a low to intermediate mutational burden, with few bona fide MHC-presented epitopes [14]. Indeed, bioinformatic analysis of TCGA data revealed that only 12% of HGSOC cases had a 90% likelihood of harboring at least one authentically processed and presented neoantigen, versus 51% of lung cancers [25]. The analysis further showed that HRD tumors (including those with BRCA1/2 mutations) exhibit higher neo-antigen load compared to HR proficient tumors [26].

In this context, an interesting example arises from a phase II study on the effect of pembrolizumab (anti-PD1) treatment in tumors with MMR deficiency, in patients with treatment-refractory progressive metastatic cancers [27,28]. A higher objective response rate was observed in patients with MMR deficiency whose somatic mutational load (detected by whole exome sequencing) was 200 fold higher than in MMR proficient patients. This hypermutated phenotype resulted in the expression of a high number of neoantigens increasing immunogenicity, thus, boosting an immune response and favoring efficacy of pembrolizumab. Interestingly, among the non-colorectal cancers included in the phase II study, were two endometrial cancer cases. Endometrial cancers have been recently classified in four molecular distinct subtypes with prognostic relevance [29] including a molecular subgroup with MMR deficiency and a subgroup with an excellent prognosis characterized by mutation of the DNA polymerase POLE involved in proofreading of DNA during DNA replication. POLE mutation causes an ‘ultra-mutated’ phenotype increasing immunogenicity and promoting a more favorable outcome, with likely sensitivity also to checkpoint inhibition-based treatment [30].

### 3.4. Reactivating the Immune System

Reactivation of inert T cells would be expected to substantially improve management of gynecological malignancies, and it is, indeed an area of active investigation. T cells activity could be regenerated by reactivating the immune system by vaccination. Neoantigens with the ability to activate an immune response can be directly identified by sequencing technology and bioinformatics pipelines [25] and exploited for vaccination therapy [31]. Within gynecological malignancies, a number of therapeutic vaccines based on the targeting of HPV E6 and E7 early proteins are under investigation supported by preclinical results [32].

### 3.5. Microenvironment and Immune Responses

The presence of an immunosuppressive environment, due to tumor-infiltrating myeloid cells, such as tumor-associated macrophages, has increasingly been recognized as impacting on ovarian cancer response to treatment. Tumor-derived factors actively recruit circulating monocytes at the tumor site where they differentiate into tumor-associated M2 macrophages promoting tumor cell proliferation and survival. Targeting immuno-suppressive M2 macrophages or activating tumoricidal M1 macrophages are attractive immunotherapeutic strategies with the potential for generating valuable anti-tumor responses [33,34].

## 4. Overcoming Roadblocks to Completing Translational Goals

Parallel breakout working groups, enriched with relevant expertise, focused on topics related to tumor tissue collection and analysis, requirements for appropriate validation of predictive biomarkers, legal and ethical issues. The aim was to develop recommendations on how to overcome potential roadblocks to facilitate the achievement of translational goals in gynecological research. The main points discussed are itemized below.

1. Pathology and biomarker research should be integral to clinical trial design to reduce the likelihood that a trial produces a negative result without mechanistic information provided.
2. Mandatory upfront central collection of pathologically reviewed patients’ tissues is to be preferred to ensure the generation of optimal trial-associated tumor biobanks with complete clinical annotation. Efforts should be made to create a harmonization program of Standard Operating Procedures (SOPs) to be shared across trial centers and across GCIG members as a set of documented guidelines taking into account issues related to sample collection, curation, and clinical data annotation.
3. When planning a collaborative translational study, two different scenarios could be hypothesized, a virtual vs. physical biobanking, each of them requiring different costs, management, and governance. In the process of selection of the most appropriate type, it should be considered that while the virtual biobanking is easy from a regulatory perspective with tissues remaining in the original sites, it needs the development of robust protocols for testing the biomarkers, as the assays will be all carried out locally. In addition, while it is useful for validation studies, the virtual biobanking is not practical for rarer tumor types or small trials.
4. Expert pathology review is recommended to ensure comprehensive assessment of gynecological cancers [35]. When considering a trial design which includes molecular tests it is recommended that translational/molecular/pathologic expertise should be consulted, to optimize sample collection and translational study design. In any case, the development of a project-specific consortium with a defined data-sharing mechanism, authorship policy and regular meetings, may substantially improve the output of the translational study.
5. A process of assay validation is required with regard to reproducibility and reliability between laboratories (use of different/multiple platforms across different labs), to validate fit-for-purpose assay platforms, analysis tools, shared controls, and cut points that should be used in the final setting assay.
6. As new analyses are developing over time, studies with molecular end-points should include as part of the trial, pathology-reviewed collection of additional biospecimen(s) for future use, for assay revalidation, and assessment of new or improved biomarkers.
7. Biomarker validation and development are related processes that should be made public and transparent at all points by all stakeholders (including industry) to ensure rapid progress with positive outcomes for patients. The early phase studies should include all-comer, non-randomized patients, assessing intra and inter patient heterogeneity (temporal and geographic heterogeneity). In early validation studies, evaluation of the full spectrum is preferable to avoid loss of information about the possible treatment effect in the biomarker-negative population, these studies can be single arm and relatively small. Progression toward validation should be based on randomization, focused on the population of interest, with adequate power for appropriate treatment/outcome interaction analyses.
8. Patient consent has to be specific and explicit, referring to future relevant research. However, to improve its acceptance, it should be a one-time consent, remaining broad but yet not unlimited in its terms. The opportunity to introduce a tiered consent has been considered. Potential exemption of consent should be considered (Declaration of Helsinki, art 32) with the development of an ethically defensible plan.
9. Engagement of multiple trial groups with the aim of streamlined tissue donation and curation should be advocated, to increase the value/impact of trials through translational research. This is particularly aimed at ending the waste of funding and effort associated with trials which fail to reach their primary or secondary endpoints

Along with these suggestions, important methodological issues still need to be solved.

- Standardization of the process of selection and optimization of the cutoff point for continuous biomarkers.
- In addition to scientific validity, the cost-benefit impact of biomarkers should also be considered. Whenever possible, based on anatomic feasibility, collection of post-progression biopsies should always be included in patient consent forms. Since this procedure is often restricted due to lack of funding, parallel institutional or trial group protocols and funding should be made available to ensure this collection.
- Issues related to the heterogeneity of the tumor and of the host (ethnic/racial variation) need to be considered.
- The use of an agreed governance document for Material Transfer is important to simplify institutional intellectual property agreements and to reduce delay.
- The creation of a big data consortium for clinical trial data to overcome the limitations related to the poor diffusion of negative results and to the different regulatory issues existing among the different entities involved should be considered.

## 5. Conclusions, Overall Goals and Key Future Tasks

The GCIG–Translational Research brainstorming day provided the opportunity for international discussion and consensus on important issues related to the inclusion of translational end-points into clinical trial designs for gynecological malignancies. Standardization and optimization of procedures for such molecular analyses are, therefore, needed, and summarized below, to maximize patients’ benefit.

- Development of a harmonization program of SOPs in a set of documented trial-specific guidelines taking into account issues related to sample collection and curation (pathological definitions and evaluation, clinical data annotation), and biomarker validation (analysis tools, choice of cut-points, impact of geographical and temporal heterogeneity, platforms, laboratory variation, etc.)
- Improve data sharing, transparency (e.g., components of biomarker signature/algorithms) publications (including negative findings) and ensure funding for sample collection. Facilitate development of consortia with a constitution, a data sharing mechanism and authorship policy that might also improve translational research funding success.
- GCIG guidelines/templates for patients’ informed consent including incorporation of global GCIG Material Transfer Agreement for widespread use to ensure streamlined access to patient specimens for the purpose of translational research.
- Increase involvement of advocates and improve reporting of translational data in a forum accessible to patients.

## Figures and Tables

**Table 1:** Biomarker applications.

Type of Biomarker	Question Addressed
Diagnostic	Cancer type/subtype identification
Prognostic	Cancer outcome definition
Predictive	Probability of response to a given drug
Pharmacodynamic	Definition of the optimal dose for efficient target engagement

**Table 2:** Biomarkers definition.

	Aims	Requirements
**Integral**	Used for patient selection within a particular trial; its detection determines patient treatment.	Assessment has to be performed in a CLIA environment; it may require IDE and additional FDA approval.
**Integrated**	Used for patient/tumor characterization; it should provide evidence of function/pathway alteration.	CLIA environment recommended; IDE is not required.
**Exploratory**	Descriptive biomarkers; used to explore other patient characteristics useful for alternative treatment(s).	No particular requirements to be performed.

Abbreviations; CLIA: Clinical Laboratory Improvement Amendments; IDE: Investigational Device Exemption.

**Table 3:** Biomarkers credentials.

	Biomarker-Enrichment Design	Biomarker-Stratified Designs	Adaptive Designs (Randomized Phase II/III Trial Design)
**Design description**	• Biomarker positive subgroup is tested. • Control/standard therapy arm controls for marker prognostic effects.	• Randomize for treatment both positive and negative patients. • Several different testing strategies are possible.	• Combine phase II and phase III trials in a phase II/III trial. • It can be used without enrichment or be stratified by biomarker
**Suitable choice when**	• Solid knowledge of biomarker biology. • Convincing evidence that the benefits of treatment are limited to the biomarker-positive subgroup.	• A clinically significant effect in biomarker-negative patients cannot be ruled out.	• The treatment benefit should be definitively assessed; • It allows a decrease of the time and number of patients required from early to later phase development.
**Major issues:**	• It does not provide information on the biomarker-negative population (off-target effects, multiple pathway targeting) • Advantages coming from biomarker refinement within trial are limited to biomarker-positive patients.	• They maintain a good statistical power even in the case of a homogeneous treatment effect across subgroups. • They provide evidence of treatment benefit in both positive and negative population. Interim analysis can guide the accrual of biomarker-negative subgroup.	Critical are • the choice of the intermediate endpoint • the criteria (error rates, timing of analyses) defining a promising activity for phase II • accrual suspension while awaiting phase II data to mature.
